# Nerve Growth Factor (NGF) as Partaker in the Modulation of UV-Response in Cultured Human Conjunctival Fibroblasts

**DOI:** 10.3390/ijms23116337

**Published:** 2022-06-06

**Authors:** Graziana Esposito, Bijorn Omar Balzamino, Maria Luisa Rocco, Luigi Aloe, Alessandra Micera

**Affiliations:** 1 Research and Development Laboratory for Biochemical, Molecular and Cellular Applications in Ophthalmological Science, IRCCS—Fondazione Bietti, 00198 Rome, Italy; graziana.esposito@fondazionebietti.it (G.E.); bijorn.balzamino@fondazionebietti.it (B.O.B.); 2 Institute of Cell Biology and Neurobiology, CNR, 00143 Rome, Italy; marialuisarocco@virgilio.it; 3 Fondazione IRET, 40064 Bologna, Italy; luigi.aloe@cnr.it; 4 Associazione NGF ONLUS, 00172 Rome, Italy

**Keywords:** NGF, ultraviolet radiation, conjunctival fibroblasts, neuroprotection, para-inflammation, epigenetics, in vitro

## Abstract

Corroborating data sustain the pleiotropic effect of nerve growth factor (NGF) in the protection of the visual system from dangerous stimuli, including ultraviolet (UV). Since UV exposure might promote ocular surface changes (conjunctival inflammation and matrix rearrangement), as previously reported from in vivo studies sustaining some protective NGF effects, in vitro cultures of human conjunctival fibroblasts (FBs) were developed and exposed to a single UV exposure over 15 min (0.277 W/m^2^), either alone or supplemented with NGF (1–10–100 ng/mL). Conditioned media and cell monolayers were collected and analyzed for protein release (ELISA, ELLA microfluidic) and transcript expression (real-time PCR). A specific “inflammatory to remodeling” pattern (IL8, VEGF, IL33, OPN, and CYR61) as well as a few epigenetic transcripts (known as modulator of cell differentiation and matrix-remodeling (*DNMT3a, HDAC1, NRF2* and *KEAP1*)) were investigated in parallel. UV-exposed FBs (i), showed no proliferation or significant cytoskeleton rearrangement; (ii), displayed a trkA^NGFR^/p75^NTR^ phenotype; and (iii), synthesized/released IL8, VEGF-A, IL33, OPN, and CYR61, as compared to unexposed ones. NGF addition counteracted IL8, IL33, OPN, and CYR61 protein release merely at lower NGF concentrations but not VEGF. NGF supplementation did not affect *DNMT3a* or *HDAC1* transcripts, while it significantly upregulated *NRF2* at lowest NGF doses and did not change *KEAP1* expression. Taken together, a single UV exposure activated conjunctival FBs to release pro-inflammatory/fibrogenic factors in association with epigenetic changes. The effects were selectively counteracted by NGF supplementation in a dose-dependent fashion, most probably accountable to the trkA^NGFR^/p75^NTR^ phenotype. Further in vitro studies are underway to better understand this additional NGF pleiotropic effect. Since UV-shield impairments represent a worldwide alert and UV radiation can slowly affect ocular surface homeostasis (photo-ageing, cataract) or might exacerbate ocular diseases with a preexisting fibrosis (pterygium, VKC), these findings on NGF modulation of UV-exposed FBs might provide additional information for protecting the ocular surface (homeostasis) from low-grade long-lasting UV insults.

## 1. Introduction

The ocular surface is covered by tear film, a multifunctional layer keeping the underneath tissue away from outdoor environment noxious agents (light, dry-climate, pollution) [1,2]. An impaired tear film composition might be responsible for failure in the natural protective function [3]. Even slow and long-lasting, ultraviolet radiations (UV) can elicit a tissue response by triggering innate immune response, p53-mediated cell apoptosis/senescence, and specific release of early oxidative stressors followed by release of chemokines/cytokines, growth factors, and other soluble mediators by epithelial cells with autocrine/paracrine effect [4,5,6]. In experimental models, UV exposure was associated with severe corneal changes, infiltration of immune cells (neutrophils), release of proinflammatory cytokines/chemokines (interleukins IL1, IL6 and IL8), increase of vascular endothelial growth factor (VEGF) for new vessel formation, and the release of oxidative stress products, all from reactive epithelial cells, fibroblasts (FBs), and resident and/or infiltrating immune cells [7,8,9,10,11,12,13,14]. UV exposure was also associated with extracellular matrix (ECM) remodeling, an effect sustained by the release of profibrogenic factors, matrix-makeover molecules, and immune-modulating cytokines (TGFβs), and the recently recognized “early danger signal” IL33 [7,12,15]. Recently, some studies highlighted the effect of osteopontin (OPN), an extracellular glycoprotein with a variety of cellular (activation, chemotaxis, and apoptosis) and matrix-remodeling activities, sustaining neuroprotective and reparative effects with the regulation of intercellular signaling, as observed in UV-exposed human skin fibroblasts by the ECM-associated cysteine-rich angiogenic inducer 61 (CYR61) protein [16,17,18].

Epigenetic factors are key mediators of UV-driven tissue remodeling and fibrosis, as supported by their implication in DNA methylation, histone modifications, and non-coding RNA modulation of fibrogenic targets [2,19,20]. Particularly, studies on cultured FBs highlighted the DNA methyltransferase 3 alpha (*DNMT3a*) and the histone deacetylase 1 (*HDAC1)* modulatory abilities on cell proliferation throughout the Kelch-like ECH-associated protein 1 (*KEAP1*) and the nuclear factor erythroid 2-related factor 2 *(NRF2)* signal proteins [21,22,23].

Highly involved in the homeostasis of the visual system, the nerve growth factor (NGF), the tyrosine kinase trkA^NGF^, and the pan-neurotrophin p75^NGF^ receptors have been recently reported to have multiple effects in UV-exposed tissues and cells [24,25,26,27,28]. Since NGF has been proven as (i), protective and therapeutic for damaged corneal and conjunctival epithelial cells; (ii), profibrogenic for corneal and conjunctival fibroblasts/myofibroblasts; and (iii), neuroprotective for retinal cells [10,11], the aim of this study was to explore the effect of NGF supplementation in UV-exposed conjunctival FBs by analyzing a few selected targets (inflammatory and epigenetic factors) previously linked to UV-exposed cells.

## 2. Results

First, the appropriate in vitro UV ray intensity was assessed starting from studies reported by others, with the aim to avoid an apoptotic and/or cell death response (sub-cytotoxic effect) [29,30,31].

**Figure 1:**
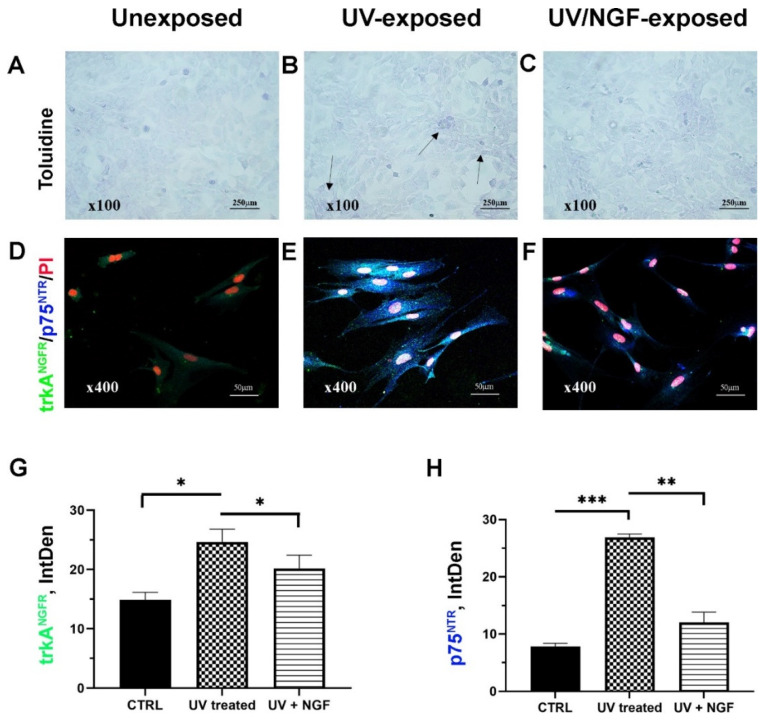
Cell morphology and NGF receptors. Light (toluidine) and confocal (immunofluorescence) images of conjunctival monolayers after 24 h of culturing as: unexposed (**A**,**D**); UV-exposed (**B**,**E**), and UV/NGF-exposed ((**C**,**F**); 10 ng/mL NGF). Detached cells were less than 10% in UV-exposed monolayers (see arrows, (**B**) vs. (**A**)), and no difference in monolayer appearance was observed in UV/NGF-exposed in comparison to unexposed ones ((**C**) vs. (**A**)). Representative confocal images display a strong trkA^NGFR^ (green) and rather absent p75^NTR^ (blue) immunoreactivity in unexposed conjunctival fibroblasts (**D**). Both trkA^NGFR^ and, particularly, p75^NTR^ immunostainings were increased in UV-exposed cells as compared to untreated ones ((**E**) vs. (**D**)). Low trkA^NGFR^ immunoreactivity was observed in UV/NGF-exposed cells with respect to untreated ones, in comparison to p75^NTR^ immunoreactivity ((**F**) vs. (**D**)). Nuclear staining: propidium iodide (PI/red). Magnifications: ×100 (**A**–**C**), ×400 (**D**–**F**). Integrated densitometric analysis (Image J) was carried out on single channel trkA^NGFR^ (**G**) and p75^NTR^ (**H**) immunostainings. CTRL, unexposed monolayers. ANOVA followed by Tukey–Kramer post hoc analysis highlighted the significant effects indicated in the graphics (* *p* < 0.05, ** *p* < 0.01 and *** *p* < 0.001) with respect to CTRL.

Serum-starved monolayers were analyzed at 3 and 24 h from UV exposure (sister cells; Figure 1A–C). Less than 10% of detached cells were quantified in UV/NGF-exposed monolayers (Figure 1C) with respect to UV-exposed (Figure 1B) and unexposed (CTRL, Figure 1A) ones. No significant changes were observed between UV- and UV/NGF-treated monolayers for cell proliferation (data not shown). On the contrary, a cell-elongated morphology was observed in UV (Figure 1E) and UV/NGF (10 ng/mL NGF; Figure 1F) optic fields, as compared to unexposed ones (Figure 1D). This elongated appearance was not associated to α-smooth muscle actin (αSMA) expression (data not shown). On the contrary, an increased immunoreactivity for p75^NTR^ was observed in UV-exposed monolayers, either in the absence (Figure 1E) or presence (Figure 1F) of NGF supplementation, with respect to unexposed monolayers (Figure 1D) and in line with previous studies [11]. Integrated densitometric analysis (Image J) was carried out (see Figure 1G–H), sustaining the low p75^NTR^ immunoreactivity with respect to trkA^NGFR^ in unexposed FBs and a reorganization upon UV and UV/NGF exposure (blue vs. green IntDen signals).

From low to undetectable levels of IL8 (ELLA microfluidics) and VEGF (ELISA) were detected in conditioned media from unexposed conjunctival FBs. After 24 h from UV exposure, different levels of IL8 (10.84 ± 0.53 pg/µg) and VEGF (0.27 ± 0.05 pg/µg) were quantified in the conditioned media as compared to unexposed ones. Physiological levels of NGF were as previously reported [11], and an increased *NGF*mRNA expression was observed in these UV-exposed cells (4.11 ± 0.21 fold change (FC-2log-scale); *p* < 0.05), encouraging our in vitro supplementation.

### 2.1. Changes in IL8 and VEGF Levels in the Conditioned Media from UV and UV/NGF Exposures

**Figure 2:**
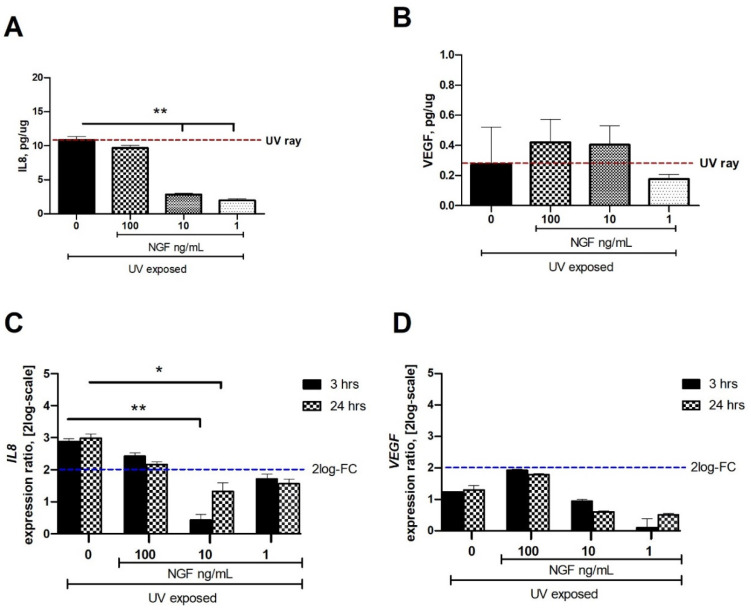
Effect of NGF supplementation on IL8 and VEGF protein and mRNA expression in UV-exposed conjunctival fibroblasts. Starved monolayers were UV-exposed either alone (0.05% FBS medium) or supplemented with NGF (1*–*10*–*100 ng/mL). Conditioned media (**A**,**B**) were collected after 24 h and analyzed ((**A**); IL8/ELLA, (**B**); VEGF/ELISA). UV-exposed conjunctival FBs showed an increase in the cytokines and growth factor content in conditioned media, as compared to unexposed ones. RNA cell extracts collected at 3 h and 24 h from exposure showed an increased expression of transcripts for *IL8* (**C**) and *VEGF* (**D**), as compared to UV-exposed ones, all calculated with respect to unexposed ones (REST analysis). Data represent mean ± SEM for all groups. REST-ANOVA followed by Tukey–Kramer post hoc analysis highlighted the significant effects indicated in the graphics (* *p* < 0.05 and ** *p* < 0.01) with respect to UV exposure; red dashed lines pointing at UV exposure and blue dashed lines for 2log-FC PCR biological significance.

IL8 protein was significantly reduced in the conditioned media from UV-exposed cells when NGF was supplemented at 1 and 10 ng/mL doses (Figure 2A). No difference was observed at 100 ng/mL NGF supplementation, while the maximum effect was observed at 1 ng/mL NGF (*p* < 0.01; Figure 2A). Changes in VEGF protein expression were observed only at 1 ng/mL NGF supplementation, as compared to the UV-exposed one (Figure 2B).

At 3 and 24 h from exposure, the related target gene expression (FC-2log-scale) was as follows: *IL8* (2.87 ± 0.38 and 2.98 ± 0.32 FC-2log-scale) and *VEGF* (1.22 ± 0.47 and 1.30 ± 0.24 FC-2log-scale).

A significant reduction of mRNA expression specific for *IL8* (C) and *VEGF* (D) was observed in UV-exposed monolayers at 3 h and 24 h supplemented with 1 ng/mL, 10 ng/mL, and 100 mg/mL NGF, as compared to UV-exposed ones. No difference or a slight upregulation was observed in UV/NGF-exposed FBs (100 ng/mL NGF), as compared to UV-exposed ones.

### 2.2. Changes in IL33, OPN, and CYR61 Matrix-Associated Proteins upon Exposures

**Figure 3:**
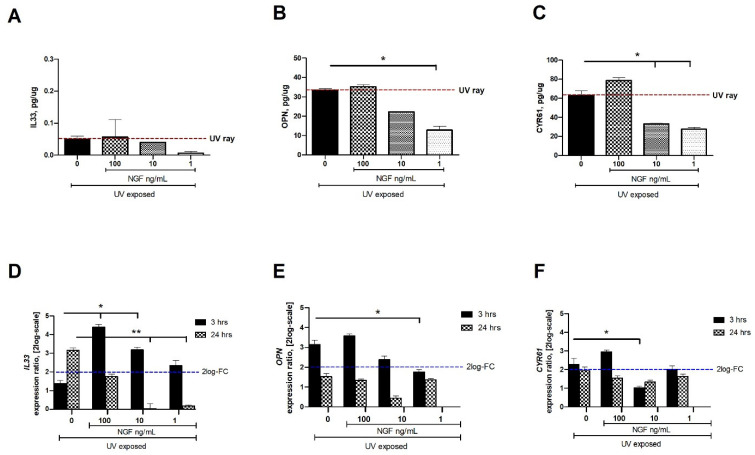
NGF supplementation affects IL33, OPN, and CYR61 expression in UV-exposed conjunctival FBs. Starved monolayers were UV-exposed either alone (0.05% FBS medium) or supplemented with increasing NGF doses (1*–*10*–*100 ng/mL). At specific time-points from exposure, conditioned media (**A**–**C**) and monolayers (**D**–**F**) were collected and analyzed (IL33, OPN, CYR61). UV exposure resulted in great release of cytokines and growth factor in conditioned media from UV-exposed conjunctival FBs, as compared to unexposed ones. The analysis of transcripts showed an increased mRNA expression for *IL33* (**D**), *OPN* (**E**), and *CYR61* (**F**) in UV exposed conjunctival fibroblasts at 3 and 24 h from exposure. NGF supplementation (1*–*10*–*100 ng/mL) counteracted this target gene expression. Data represent mean ± SEM for all groups. REST-ANOVA followed by Tukey–Kramer post hoc analysis highlighted the significant effects indicated in the graphics (* *p* < 0.05 and ** *p* < 0.01), with respect to UV exposure; red dashed lines pointing at UV exposure and blue dashed lines for 2log-FC PCR biological significance.

Low to undetectable levels of IL33, OPN, and CYR61 (ELLA) were detected in conditioned media from unexposed conjunctival FBs. After 24 h from UV exposure, changes in IL33 (0.05 ± 0.01 pg/µg), OPN (33.34 ± 0.83 pg/µg), and CYR61 (63.06 ± 4.68 pg/µg) protein content were quantified in conditioned media, as compared to unexposed ones. The levels of IL33, OPN, and CYR61 proteins were significantly reduced in the conditioned media from UV/NGF-exposed cells (1 and 10 ng/mL NGF; Figure 3A,C). No difference was observed at the higher NGF concentration (100 ng/mL; *p* > 0.05), while the maximum effect was detected at the lowest dose (1 ng/mL NGF; *p* < 0.01). Changes in target gene expression were observed at 3 h and 24 h as follows: *IL33* (1.37 ± 1.43 and 3.16 ± 1.33 FC-2log-scale), *OPN* (3.15 ± 0.37 and 1.53 ± 0.34 FC-2log-scale), and *CYR61* (2.27 ± 0.45 and 1.95 ± 0.35 FC-2log-scale). Upon NGF supplementation, a significant reduction of mRNA expression was detected as displayed for *IL33* (D), *OPN* (E), and *CYR61* (F) in UV/NGF-exposed monolayers at 3 h and 24 h if supplemented with 1 ng/mL and 10 ng/mL NGF, as compared to UV-exposed ones. No difference or a not significant upregulation was observed for these transcripts at the higher NGF concentration (100 ng/mL NGF; UV/NGF-exposed vs. UV-exposed extracts; *p* < 0.05).

### 2.3. DNMT3a, HDAC1, NRF2, and KEAP1 Transcript Expression upon Exposures

**Figure 4:**
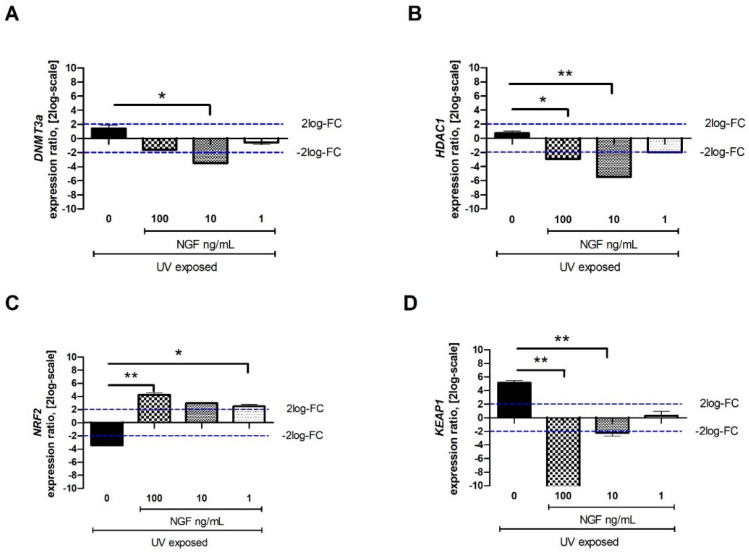
Effect of NGF supplementation on specific tissue remodeling-associated epigenetic targets. Starved monolayers were UV-exposed either alone (0.05% FBS medium) or supplemented with increasing NGF doses (1*–*10*–*100 ng/mL). After specific exposure, conditioned media were collected, and monolayers were extracted for total RNA analysis. UV exposure resulted in slight transcripts for *DNMT3a* (**A**), no transcript modulation for *HDAC1* (**B**), low transcripts for *NRF2* and high transcripts for *KEAP1* (**D**). NGF supplementation (1*–*10*–*100 ng/mL) downregulated the expression of *DNMT3a* (**A**), *HDAC1* (**B**), and *KEAP1* (**D**) at all concentrations tested. *NRF2* upregulation was significant at all NGF doses (**C**). Data represent mean expression ratio ± SEM (fold changes as 2log-scale). REST analysis followed by ANOVA—Tukey–Kramer post hoc analysis defined significant transcript changes as indicated in the graphics (* *p* < 0.05 and ** *p* < 0.01, with respect to UV exposure); blue dashed lines pointing at 2log-FC biological relevant limit.

Few epigenetic genes known to regulate fibrogenic functions (*DNMT3a, HDAC1, NRF2*, and *KEAP1*) were investigated after UV exposure. A significant downregulation of mRNA expression was observed for *DNMT3a* (Figure 4A) and *HDAC1* (Figure 4B) in UV/NGF-exposed FBs at all concentrations tested. As shown in Figure 4C, a significant decrease of *NRF2* transcript was detected in UV-exposed FBs, while an increase was found in UV/NGF-exposed FBs at 1 ng/mL NGF and 10 ng/mL NGF (*p* < 0.05), highly significant at 100 ng/mL NGF (*p* < 0.01). As displayed in Figure 4D, *KEAP1* transcript expression was significantly downregulated upon UV exposure and quite downregulated at the lowest NGF supplementation (1 ng/mL), significantly downregulated in a dose-dependent fashion (*p* < 0.01).

## 3. Discussion

**Figure 5:**
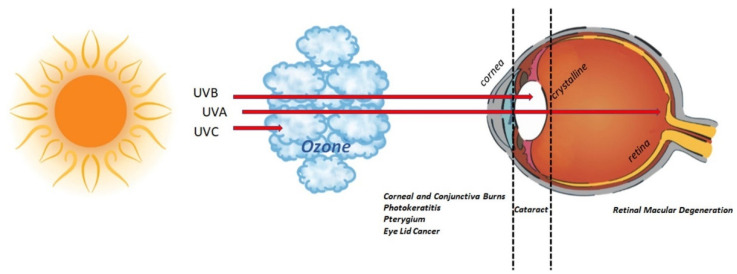
UV radiation and eye. Schematic representation of UV route inside the eye. Sunlight is a natural source of UV, accountable for 10% of UV rays (including UV-B, 290 to 320 nm) [32]. As depicted in the graphical representation, UV-C (280 nm) are absorbed by the ozone in the atmosphere; UV-B rays (280*–*315 nm) are completely absorbed by corneal structure and crystalline; UV-A rays (315*–*400 nm) are partially absorbed by the cornea, while the remainder can reach the retina. Exposure to UV rays might insult the visual system or lead to/exacerbate an existing chronic status (pterygium, cataract, retinal macular degeneration). The major eye disorders associated with UV ray exposure are listed below the eyeball.

The ocular surface is continuously exposed to sunlight UV depending on geographical position, climate, lifestyle, and working habits [1,2,3,32,33,34]. Since UV rays can end at the ocular surface, the conjunctiva might be an elective target for an undesired development of inflammation and/or tissue remodeling in predisposed subjects (Figure 5). The possibility that even a single UV exposure can induce an “alert response” in conjunctival FBs, with the release of a specific protein profile, cannot be excluded, as prospected in previous studies [2,34]. Multiple linked events have been observed in cultured UV-exposed FBs, including the release of chemokines, cytokines, growth/angiogenic factors, and several profibrogenic mediators [2,34]. Since UV-exposed FBs might affect the conjunctival microenvironment and epithelia-stromal crosstalk and NGF can exert pleiotropic effects at the ocular surface [11,27,35], a possible NGF effect in counteracting UV-protein signature was prospected and investigated. A single UV insult with no apoptotic or necrotic response (sub-cytotoxic effects) was tested.

First, our UV-exposed monolayers did not show cell proliferation, except for a small number of detached and/or dead cells. The elongated-shape morphology resulting from UV exposure was not associated with a consistent αSMA/actin-cytoskeletal reorganization, suggestive of no FB differentiation. In addition, the expression of the pan-neurotrophic p75^NTR^ receptor after UV exposure suggests that both UV and UV/NGF cell phenotypes can respond to NGF [35,36].

As reported, UV exposure can prompt an in vitro cellular response characterized by the release of different inflammatory and fibrogenic factors (IL1β, IL6, IL8, NGF, VEGF, IL33, OPN, CYR61) usually unexpressed or faintly expressed in normal conditions [12,15,16,37,38,39]. Herein, parallel experiments were carried out to compare these expressions between UV- and UV/NGF-exposed human conjunctival FBs. Our in vitro system showed the synthesis and release of IL8, VEGF, IL33, and CYR61 upon UV exposure, in line with previous studies [12,15,16,17,18,37,38,39]. Since the release of IL8 from UV-exposed monolayers was not observed with NGF supplementation, the possibility of a NGF involvement in preserving a low-grade inflammation can be prospected, as IL8 is a well-known chemoattractant for neutrophils [7,9,37,38,39]. The observation on VEGF levels in UV- and UV/NGF-exposed monolayers might find an explanation in previous studies on NGF–VEGF interaction [13]. The increased expression of IL33, OPN, and CYR61 upon UV exposure was found offset at lower NGF supplementations, suggesting an involvement of NGF in ECM metabolism. In previous studies, the release of both NGF and VEGF by epithelial cells was associated with new vessel formation, cytokines’/chemokines’ pathway activation (IL1, IL6, and IL8), and recruitment of immune cells (neutrophils), resulting in the maintenance of corneal transparency, as described in UV-induced corneal inflammation [40,41,42]. In other studies, UV insults were associated with stromal cells’ activation and tissue remodeling, representing an aspect of great importance in the case of ocular surface disorders characterized by fibrotic processes [29,30,37,38,39,41,42].

Certainly, epigenetic regulations of fibrogenic processes are strictly linked to genetic background, environmental factors, and other issues, including gender and ageing [1,2,3,4,34,43]. Since noxious environmental factors can activate epigenetic factors, the investigation of DNA, histones, or non-coding RNAs might be useful for the identification of potential targets for prevention and/or therapy [2,44,45]. As reported, DNA methylation patterns are established by the de novo *DNMT3a/3b*–*HDAC1* synthesis and subsequently maintained by *DNMT1* [46,47,48]. Our findings on *DNMT3a* and *HDAC1* transcript deregulation upon UV/NGF exposure might support the counteracting effect of NGF on the release of profibrogenic cytokine by UV-exposed FBs. Furthermore, the low *KEAP1* and high *NRF2* expressions detected in UV/NGF-exposed FBs might imply the activation of *NRF2/KEAP1* axis with modulatory roles on tissue remodeling and fibrosis [23,49]. All of these biomolecular aspects are of great importance, as epigenetic modifications are reversible and, therefore, suitable “prime targets” for therapeutic intervention [50,51].

Most of the NGF effects on UV-exposed FBs occurred mainly at the lower NGF concentrations, suggesting the contribution of the high affinity trkA^NGFR^/p75^NTR^ receptor [11,26,52]. This aspect is not surprising, as encouraged by previous studies highlighting the survival role of the high-affinity NGF receptor in tissue homeostasis [28,36]. To support this, UV exposure affected both NGF and trkA^NGFR^ expression in corneal, retinal, and cutaneous tissues, while UV/NGF exposure enhanced NGF and trkA^NGFR^ expression and reduced cell death [11,36]. As timing studies showed transcript expression at 3 and 24 h, the possibility of an endogenous production/utilization of NGF, in concert with other factors, cannot be excluded [11].

To summarize, several studies were carried out on epithelial and retinal cells, while little attention was given to healthy aged FBs. As photo-ageing is firmly associated with low-grade and long-lasting exposure, our studies privileged cultures at 5–8 passages (aged sister cells) [25,35]. Some studies reported the modulation of few pterygium-related genes in UV-exposed FBs, reinforcing the link between UV and initiation and/or progression of human pterygium and/or exacerbation of preexisting fibrosis [6,53]. In addition, ocular surface discomfort might also occur in the case of light (UV) reflected from snow, water, and sand [33].

Noteworthily, the ocular surface unit needs to be protected from any kind of insult to maintain the corneal functionality [54]. Recently, increased UV exposure due to ozone thinning has been associated with “dry eyes of environmental origin”, “urban syndrome”, exacerbations of preexisting ocular fibrosis (conjunctival pterygium, photokeratitis, climatic droplet keratopathy), and cataract-genesis, and the current generation is more greatly exposed to UV radiation than earlier ones, due to the increase in unshielded UV rays reaching the Earth’s surface [32,33,34,43,51,54,55,56,57,58]. This aspect appears of great importance, as the corneo-conjunctival limbus is also populated by mast cells with gatekeeper functions that might react to photo-exposure influencing the local microenvironment [57,58].

## 4. Materials and Methods

### 4.1. Cell Cultures and Analytical Reagents

Primary human conjunctival FBs (*n* = 3; InnoProt, Bizkaia, Spain) were expanded in complete Dulbecco’s Modified Eagle Medium (DMEM) (5th to 8th generation), and experimental plates were prepared from sister cells including a plate with UV and UV/NGF subgroups and a parallel one with the unexposed one. Since primary FBs were purchased, the study was approved by the intramural scientific committee of the IRCCS-Fondazione Bietti. Routine plasticware and analytic-grade reagents were purchased from Starlab (Milan, Italy), Euroclone (Milan, Italy), or ICN Biochemicals (Costa Mesa, CA, USA). For culturing purposes: tissue culture plasticwares were obtained from NUNC (Roskilde, Denmark); DMEM, fetal bovine serum (FBS), 1 mM glutamine and 1% pen/strep cocktail, and Hank’s Balanced Sodium Solution (HBSS) were from Euroclone. For biomolecular analysis: RNAfree MilliQ water (DirectQ; Merck-Millipore, Vimodrone, Milan, Italy) and phosphate-buffered saline (PBS: 10 mM phosphate pH 7.5 and 0.9% saline) were autoclaved and used for the experiments.

### 4.2. UV Experiments and Subgroups

Time-exposure and monolayer distance from UV source were according to previous studies [11,29,30]. Briefly, cells were grown on round-glass placed on 24-well plates in the presence of complete DMEM (10% FBS, glutamine and antibiotics), and 24 h serum-starved monolayers were exposed to UVA/B with a medical/cosmetic vertical UV source lamp (KN-4003A/BL2; Kernel, Xuzhou, China) equipped with UV bulbs (9 W PL-S 2P/01; Philips, Amsterdam, the Netherlands) providing an emission spectrum ranging between 300 and 400 nm (peak 368 ± 3 nm for UVA and 311 ± 3 nm for UVB). Irradiation was performed placing the UV source apparatus on a home-made scaffold placed under the tissue culture hood to fix the distance, avoiding any movement (working distance: 3 cm ± 0.5 cm; exposure time: 15 min; irradiance for: UVB, 0.047 W/m^2^ and UVA, 0.230 W/m^2^; total irradiance: 0.277 W/m^2^) [30,31,51]. Briefly, DMEM (0.05% FBS) alone or supplemented with NGF (1–10–100 ng/mL) was added to monolayers before UV exposure. After a single treatment exposure, both conditioned media (24 h) and monolayers (3–24 h) were collected for specific analysis.

Unexposed cells (control sister cells; no UV-exposed or NGF-supplemented) were carried out for each experimental plate set.

### 4.3. Conditioned Media and Monolayer Preparation

After UV exposure, conditioned media were quickly collected, and monolayers on round coverslips (2 × 10^5^) were washed in sterile HBSS (pH 7.5) and postfixed in 3.7% buffered PFA. In other studies, monolayers were treated with trypsin-EDTA solution, and detached single cells (10^6^ cells per well; six-well plates) were washed/harvested with 10% FBS (to neutralize enzyme activity), pelleted by centrifugation (3500 rpm/3 min), and used for RNA extraction.

### 4.4. Light and Confocal Microscopy

Cell viability, proliferation, and differentiation analysis were carried out on monolayers. After UV exposure, conditioned media were quickly removed, and monolayers on round coverslips (2 × 10^5^) were washed in HBSS (pH7.5) and postfixed in 3.7% buffered PFA. Light and confocal TIFF-converted pictures were assembled by Adobe Photoshop 7.0 (Adobe Systems Inc., San Jose, CA, USA). Magnifications were reported in figure legends and size bar in images as produced by the software.

#### 4.4.1. Toluidine Blue Staining

Staining was performed according to a standard procedure for cytological specimens (Bioptica, Milan, Italy), and randomly selected optic fields were observed and acquired with a light transmission direct Eclipse E200 microscope connected to a digital camera (×10/0.45 NA objective; ×40/0.60 NA objective; Nikon, Tokyo, Japan). Images were provided by the software (NIS elements; Nikon).

#### 4.4.2. Immunofluorescence

Postfixed monolayers were quenched in 50 mM NH_4_Cl (autofluorescence) and blocked/permeabilized in 3% BSA–0.03% Triton X-100 (TX), according to a standard procedure. Monolayers were probed with first (rabbit anti-trkA^NGFR^ and goat anti- p75^NTR^ antibodies; 4 µg/mL, R&D) and secondary (species-specific donkey raised Cy5 or FITC conjugates; 1/500 diluted in 0.03% Tween20; Jackson Immuno-research, Cambridge House, UK) antibodies. Nuclear counterstaining was performed with propidium iodide (25 µg/mL) in PBS containing RNase (20 µg/mL). Coverslips were mounted in hand-made anti-fading medium. Irrelevant isotype-matched IgG antibodies (Vector Laboratories, Inc.; Burlingame, CA, USA) were incubated in monolayers that were used as internal controls (channel series). Immunofluorescent monolayers were examined, and images were acquired using an inverted E2000-U microscope equipped with the C1 software (×20/0.45 NA objective; ×40/0.60 NA objective; Nikon).

### 4.5. Protein Analysis

Biochemical analyses were carried out on collected conditioned media quickly stabilized (1:200 protease inhibitor cocktail; Pierce Biotechnology, Rockford, IL, USA) to protect proteins from intrinsic protease digestion; centrifuged (15,000 rpm/4 °C/10 min) to remove cell death/debris; and spectrophotometrically analyzed (3 μL sample) for protein quantification (A280 software; NanoDrop N1000 Spectrophotometer; Thermo Fisher Scientific, MA, USA).

#### 4.5.1. Ella Microfluidics Platform

IL8, IL33, CYR61, and OPN were analyzed on clarified conditioned media by using the Ella microfluidics platform (Protein Simple, CA, USA). All steps for immunoassay, except sample preparation, were carried out automatically according to a standard procedure provided by the manufacturers. Cartridges (customized for the specific targets and containing lot-specific standard curves) were preloaded with 1:2 diluted conditioned media in loading buffer (Protein Simple). Single data (pg/µg) for each sample were automatically calculated (internal triplicates). Attention was devoted to data with optical density (OD) values inside the linear range of the standard curve automatically generated.

#### 4.5.2. ELISA Analysis

VEGF was measured by conventional ELISA (duo-set kit from R&D System, Minneapolis, USA, and ELISA kit from Thermo Scientific, Waltham, MA, USA). Conditioned media were prediluted (1:2) in lysis buffer supplemented with protease inhibitors (PMSF) and loaded on 96-well precoated plates. Standard curves and steps in the procedure were carried out according to the manufacturers with minor modifications. Standard curve ranges and detection limits were according to kits. Specific OD values were recorded after reading the plates at λ490 nm corrected to λ560 nm in a 96-well plate reader (Sunrise; Tecan, Männedorf, Switzerland). A polynomial third-order function (r = 0.999) was produced and used for generating concentrations.

### 4.6. Molecular Analysis: Two-Step Real Time PCR

Total RNAs were extracted according to the TRIfast technique (Euroclone); treated with DNAseI (AB1709; Ambion Inc., Austin, TX, USA); and spectrophotometrically analyzed for 260/280 ratio and RNA quantity (Nanodrop N1000 Spectrophotometer). RNA quality was verified randomly in 1% agarose gel electrophoresis (Promega, Milan, Italy) in a horizontal minicell chamber (Biorad, Hercules, CA, USA). Only samples with a 260/280 ratio > 1.8 were used for cDNA synthesis, starting from 3 µg total RNA and using the GoScript standardized procedure (Promega) including random hexamers and dNTs (Promega) in a OneCycle programmable thermocycler (Peqlab, VWR Radnor, Radnor, PA, USA). For amplifications, 3 µL cDNAs for target gene (1 µL for reference ones) was amplified in a 20 µL final volume of SYBR Green PCR mixture (Applied Biosystems, Foster City, CA, USA), using the Opticon2 real-time thermocycler (MJ Research, Watertown, MA, USA). Amplification profile was: one cycle of 95 °C/5 min initial denaturation followed by 35–40 cycles at 95 °C/30 s (denaturation), 58–61 °C/25 s (annealing temperature; AT), and 72 °C/30 s (elongation), followed by fluorescence monitoring at 60–90 °C, 0.01 °C for 0.3 s and further elongation at 72 °C/5 min. Negative and positive controls were run in parallel, according to a standard procedure. The specific pairs primers, the accession number, and the length of amplicons are shown in Table 1.

### 4.7. Statistical Analysis

Experiments were performed in triplicate, starting from *n* = 3 primary cell sets expanded/used at 5th–8th generation range, and analyzed three times to validate results. Descriptive statistics (mean ± SD in the text and mean ± SEM in the figures) and ANOVA coupled to post hoc analyses were carried out for identifying significant differences between unexposed, UV-exposed, and UV/NGF-exposed groups (Rstudio software [59]). Graphical representations were assessed using the GraphPad Prism 9.2 (GraphPad, San Diego, CA, USA). For molecular analysis, only normalized samples (run in duplicate) were amplified, and cycle threshold (Ct) values from good melting curves were used for analysis in the REST software [60]. Fold changes were calculated as the expression level of target transcript with respect to the reference one (glyceraldehyde 3-phosphate dehydrogenase, *GAPDH*), considering treated versus untreated ones (2log scale). According to the REST formula, values inside the [−2 and +2] range were not considered of biological relevance, and a blue dashed line was introduced in the graphics [60]. For significance, *p* values ≤ 0.05 were considered, and asterisks were used to label significance close to bars (* *p* < 0.05 and ** *p* < 0.01; *** *p* < 0.001).

## 5. Conclusions

Overall, the findings of this study highlight the ability of NGF to counteract the release of some proinflammatory/profibrogenic cytokines by UV-exposed FBs, extending the field of knowledge between UV, NGF, and ocular surface. Genetic background, epigenetic mechanisms, and environmental factors might contribute to the development of UV-driven ocular diseases. The downregulation of some inflammatory and epigenetic factors by NGF might provide new opportunities for the development of targeted therapies. Herein, the interest was double, as most of the chronic exposures to solar UV rays are associated with exacerbation of existing ocular fibrosis (pterygium, pinguecula, squamous metaplasia, or carcinoma) or with some environment-associated dry eye forms [32,33,53]. NGF eye drop substitute might represent, in the near future, an alternative for protecting the ocular surface from UV lights [3,15,51,58,61]. Further in vitro studies on UV-exposed conjunctival epithelial and fibroblast co-cultures (3D) are underway to better clarify the potential contribution of NGF in sub-cytotoxic UV exposure herein prospected.

## Figures and Tables

**Table 1:** Primers used in this study. Primer pairs were designed one intron-spanning specifically to produce an amplicon no longer than 150 bps (amplicon size). Gene direction sequences (For and Rev) were generated by prime3 software according to common parameters applied to specific mRNA complete sequences (www.ncbi.nlm.nih.gov (accessed on 25 June 2019), nucleotide section) for real-time PCR amplification. Amplification profile was as shown in the text.

Gene/Accession Number	Sequence (For/Rev; 5′-3′)	AT/Amplicon
IL8 BC013615	5′- TCT CTT GGC AGC CTT CCT G -3′ 5′- TGG GGT GGA AAG GTT TGG -3′	59 °C/116 bps
VEGF AF022375	5′- CTC CGT AGT AGC CGT GGT CT -3′ 5′- CCC CTC TCC TCT TCC TTC TC -3′	61 °C/131 bps
IL33 AY905581	5′- TGA GTC TCA ACA CCC CTC AA-3′ 5′- AAG ACA AAG AAG GCC TGG T-3′	59 °C/136 bps
OPN J04765	5′- GAA ACC CAC AGC CAC AAG C -3′ 5′- CTG TGG AAT TCA CGG CTG AC -3′	60 °C/139 bps
CYR61 BC009199	5′- CAC CCT TCT CAC TTG ACC-3′ 5′- CGT TTT GCT GCA GTC CTC-3′	59 °C/106 bps
DNMT3a C032392	5′-GCA CTC AAG GGC AGC AGA TA-3′ 5′-TTC CAG GCT TCC AGG GTT AG-3′	59 °C/129 bps
HDAC1 U50079	5′- GGG ATC GGT TAG GTT GCT TC-3′ 5′- AGG GCC ACA GCT GTC CTC ATA-3′	59 °C/100 bps
NRF2 BC011558	5′- ACA CGG TCC ACA GCT CAT C-3′ 5′- TGC CTC CAA AGT ATG TCA ATC A-3′	58 °C/107 bps
KEAP1 BC015945	5′- GGG TCC CCT ACA GCC AAG-3′ 5′- TGG GGT TCC AGA AGA TAA GC-3′	58 °C/106 bps
NGF V01511	5′- CTG GCC ACA CTG AGG TCG AT-3′ 5′- TCC TGC AGG GAC ATT GCT CTC-3′	53 °C/120 bps
GAPDH BC013310	5′-GAA GGG GTC ATT GAT GGC AAC-3′ 5′-GGG AAG GTG AAG GTC GAG AGT C-3′	60 °C/111 bps

Legend: AT, annealing temperature; bps, amplicon length; For, forward (above); Rev, reverse (below).

## Data Availability

All data relevant to the study are included in the article or uploaded as supplemental information.
